# Physiologic dentin regeneration: its past, present, and future perspectives

**DOI:** 10.3389/fphys.2023.1313927

**Published:** 2023-12-11

**Authors:** Myungjin Lee, Yoon Seon Lee, Won-Jun Shon, Joo-Cheol Park

**Affiliations:** ^1^ Department of Conservative Dentistry, School of Dentistry, Dental Research Institute, Seoul National University, Seoul, Republic of Korea; ^2^ Laboratory for the Study of Regenerative Dental Medicine, Department of Oral Histology-Developmental Biology, School of Dentistry and Dental Research Institute, Seoul National University, Seoul, Republic of Korea

**Keywords:** dentin-pulp complex, odontoblasts, dentinal tubules, tertiary dentin, dentin regeneration

## Abstract

Regenerative dentistry has rapidly progressed since the advancement of stem cell biology and material science. However, more emphasis has been placed on the success of tissue formation than on how well the newly generated tissue retains the original structure and function. Once dentin is lost, tertiary dentinogenesis can be induced by new odontoblastic differentiation or re-activation of existing odontoblasts. The characteristic morphology of odontoblasts generates the tubular nature of dentin, which is a reservoir of fluid, ions, and a number of growth factors, and protects the inner pulp tissue. Therefore, understanding the dynamic but delicate process of new dentin formation by odontoblasts, or odontoblast-like cells, following dentinal defects is crucial. In this regard, various efforts have been conducted to identify novel molecules and materials that can promote the regeneration of dentin with strength and longevity. In this review, we focus on recent progress in dentin regeneration research with biological molecules identified, and discuss its potential in future clinical applications.

## 1 Introduction

Dentin is a complex mineralized tissue primarily composed of hydroxyapatite crystals, collagen fibers, and a fluid-filled tubular structure that extends from the pulp to the dentino-enamel or dentino-cementum junctions. Dentin is formed by highly specialized cells called odontoblasts, which secrete an extracellular matrix comprising collagen fibers and non-collagen proteins that serve as a scaffold for subsequent mineralization (Goldberg et al., 2011). Odontoblasts deposit dentin throughout the life of a tooth, albeit at a slower rate after early dentinogenesis, contributing to the thickening of dentin and potentially aiding in response of the tooth to external insults. As a consequence of dentinal injury or decay, tertiary dentinogenesis of two different natures occur to protect and maintain dental pulp integrity (Magloire et al., 1992; Smith et al., 2003). When secretory activities of quiescent odontoblasts are re-activated, reactionary dentin that is structurally and functionally similar to physiologic dentin is formed. On the other hand, newly differentiated odontoblast-like cells form pathologic reparative dentin, which is often less organized and more akin to bone-like tissue rather than dentin at the histological level. Likewise, physiologic dentin regeneration and pathologic dentin repair are two distinct processes aimed at restoring dentin functionality following damage, and re-establish a protective barrier for the pulp, alleviate sensitivity, and prevent further loss.

Physiologic dentin regeneration, in particular, seeks to recreate dentin that closely mimics its original, healthy state. This includes the reconstruction of dentinal tubules that integrate seamlessly with remaining dentin, the restoration of dentin-pulp complex, and the engagement of cellular and molecular pathways that govern dentinogenesis. The unique structure and function of true dentin, characterized by its distinctive tubular architecture housing odontoblasts and nerve endings, not only confers mechanical resilience to the tooth but plays a crucial role in the tooth’s immune and sensitivity responses. A number of biological molecules that can direct the odontoblastic differentiation of dental pulp cells have been studied, most of which are part of key signaling pathways regulating dentinogenesis during tooth development (Thesleff, 2003). These include TGF-β, BMP and Wnt/β-catenin signaling known to orchestrate the complex processes of cell differentiation, matrix deposition, and mineralization. The use of bioactive molecules in dentin regeneration has emerged as a promising approach, leveraging the biological cues to promote natural tissue regeneration. It is based on the concept that the application of bioactive molecules can either efficaciously re-activate underlying odontoblasts after mild stimuli or promote progenitor cell migration from pulp to the injured site, followed by proliferation and differentiation after severe stimuli. This literature review synthesizes key findings from various studies focusing on the application of bioactive molecules for the regeneration of dentin within which the tubular structure is well preserved.

### 1.1 Early research on dentin matrix proteins and growth factors

In addition to depositing the type 1 collagen-rich matrix, ofdontoblasts secrete many different bioactive molecules that are ultimately sequestered in the dentin and predentin as mineralization progresses. They are collectively called “dentin matrix proteins,” and are released when dentin undergoes demineralization in carious condition or in various clinical procedures (Kim et al., 2012). Dentin matrix proteins include growth factors and cytokines, other non-collagenous proteins, neuropeptides and neurotrophic factors, glycosaminoglycans, and serum/plasma proteins. Once liberated, these molecules act locally on the underlying odontoblast layer and stimulate the secretory activity of the odontoblasts, resulting in reactionary dentin. Under a situation where the external stimuli are so pervasive that it leads to cell death, dentin matrix-derived molecules act on the potential stem cell sources inside the pulp and induce them to migrate, proliferate, and differentiate into odontoblast-like cells. Newly differentiated odontoblast-like cells deposit reparative dentin.

The effects of non-collagenous dentin matrix proteins on dentin regeneration have been extensively investigated in many *in vivo* studies. Treatment of EDTA-soluble preparation of dentin matrix proteins (ESDP) in the ferret cavity model resulted in the deposition of reactionary dentin retaining dentinal tubule structures continuous with those of the remaining dentin (Smith and Leaver, 1979; Smith et al., 1994). The dentinogenic potential of ESDP was further confirmed by an *in vivo* study using non-human primate teeth. When ESDP was used as a cavity liner, it stimulated the formation of reactionary dentin the most among RMGIC and calcium hydroxide liner groups (Duque et al., 2006). The regenerated dentin showed characteristics of physiological dentin including the tubule structure. Dentin phosphorphoryn (DPP) is the most abundant non-collagenous protein in the dentin matrix. When DPP cross-linked to type I atelocollagen fibrils was applied in the area of pulp exposure, tubular dentin with a well-aligned odontoblast layer was formed adjacent to the remaining dentin, implicating its potential as a biocompatible pulp capping agent (Koike et al., 2014). On the other hand, when demineralized bone matrix protein (DBM) was used as direct pulp capping material in a rat pulpotomy model, more regular and thinner reparative dentin bridge was formed than when calcium hydroxide was used. Nevertheless, no tubular structure was observed in either group (Liu et al., 2017). Presumably, components of the bone matrix differ from those of the dentin matrix, which is a reservoir of bioactive molecules that can trigger formation of physiologic tubular dentin.

Transforming growth factor (TGF)-β, a member of transforming growth factor superfamily, has been one of the most extensively studied non-collagenous dentin matrix proteins in terms of dentin regeneration. Three isoforms (β1- β3) present in the dentin matrix can participate in tissue repair by promoting mineralization and odontoblast differentiation (Cassidy et al., 1997; Sloan and Smith, 1999). ESDP treated with TGF- β1 neutralizing antibody lost its inductive activity of odontoblast differentiation *in vitro* (Begue-Kirn et al., 2004). Furthermore, canine pulp treated with the demineralized dentin matrix pre-incubated with TGF- β1 neutralizing antibody, completely failed to develop tertiary dentin, while pre-incubated native dentin matrix led to the formation of new dentin with extremely scarce and irregular dentinal tubules (Tziafas and Papadimitriou, 1998). When TGF- β1-soaked millipore filter was placed in the exposed pulp, tubular matrix formed around the filter. The dentinogenic activity of TGF-β1 was further explored in various pulp capping models. Among the growth factors (TGF-β1, EGF, PDGF, BFBF and IGFII) used as capping medications, only TGF-β1-treated group showed well-calcified dentin bridge with reparative tubule structure (Hu et al., 1998). New dentin, which formed after applying TGF-β1-loaded calcium phosphate equipped with poly microsphere, occasionally possessed tubules, although the effect of calcium phosphate scaffold itself cannot be neglected (Zhang et al., 2008). Chitosan membrane is another scaffold that succeeded in regenerating tubular dentin when loaded with TGF-β1 (Li et al., 2014). All TGF-β1-treated groups manifested clear tubule structures within the newly formed reparative dentin. These results suggest that TGF-β1 may be one of key factors that bestows dentin matrix a dentinogenic potential.

Dentinal responses to recombinant forms of four different growth factors (TGF-β1, IGF-1, bFGF, BMP-7) were further evaluated in dog’s teeth (Kalyva et al., 2010). TGF-β1 showed the greatest deposition of intratubular dentin, followed by BMP-7. While the result accorded with the previous reports that TGF-β1 induces tertiary dentinogenesis, the newly accumulated dentin showed an atubular structure. On the other hand, BMP-7 treatment led to the deposition of tubular dentin, partly conforming to the previous report that allogenic crude BMP-7 treatment onto the amputated pulp induces the formation of osteodentin by 4 weeks, and tubular dentin next to the osteodentin by 8 weeks (Nakashima, 1990). A few more studies also demonstrated that BMP-7 could stimulate tubular dentin formation, but only to a small extent underneath a substantial amount of osteodentin (Rutherford et al., 1994; Jepsen et al., 1997). After the autologous dermal fibroblasts transfected with BMP-7 were inserted into inflamed ferret pulps, reparative dentin formation was observed (Rutherford, 2001). Newly formed mineralized structure resembled normal dentin, retaining tubules, predentin and odontoblast-like cells lining the predentin-pulp interface. Whether BMP-7 can stimulate tubular dentin formation *de novo* is questionable due primarily to insufficient evidence. Nevertheless, it seems likely that pre-existing osteodentin may help create an environment that is favorable for tubular dentin formation.

Bone morphogenetic protein 2 (BMP-2), which is a constituent of the TGF-β families, and an autocrine protein, has also been shown to mediate odontoblast differentiation. BMP-2 loaded on the injectable nanofibrous microspheres was released in a controlled manner to induce odontoblastic differentiation of stem cells of apical papilla (Wang et al., 2016). No tooth model was used, but the authors remarked that nanofibrous microspheres made themselves a microenvironment for reparative dentin formation. In another study, BMP-2 was transduced to dental pulp cells using adenoviral vector in a rat dentin exposure model, and as a result, more dentinal tubules were identified in the newly formed tissue than in that of the control group (Ni et al., 2018). Further, the combined effect of BMP-2 with a novel secretory protein Nel-like molecule-1 (NELL-1) was analyzed in a rat pulp exposure model (Wu et al., 2019). The adverse effects of high BMP-2 concentration have been reported to be mitigated by NELL-1 (Shen et al., 2016). Interestingly in that context, BMP-2 exhibited a synergistic effect on reparative dentin formation with NELL-1. The newly formed dentin showed a heterogenous nature with an area composed of abundant dentinal tubules despite their short and irregular morphology.

### 1.2 Emerging molecular mechanism: Wnt activation

Wnt signaling has been reported to play a significant role during different tooth developmental stages (Tummers and Thesleff, 2009) via both canonical and non-canonical pathways. Wnt proteins are secreted, lipid-altered glycoproteins in mammalian cells with nineteen identified members. Canonical signaling pathways rely on β-catenin accumulation, while non-canonical pathways bypass β-catenin involvement. Following tooth injury, Wnt signaling has been shown to stimulate tertiary dentin formation in a number of studies. Beta-catenin overexpressing mice demonstrated enhanced accumulation of atubular osteodentin morphology, suggesting that excessive Wnt activation leads to accelerated dentin secretion (Zhao et al., 2019). Indeed, pulp capping with antagonists of glycogen synthase kinase (GSK-3β) that phosphorylates β-catenin and axin leading to their degradations resulted in a greater amount of reparative dentin than with MTA (Neves et al., 2017). Furthermore, reactionary dentin formation was enhanced in homozygous Axin2 mice dental pulp, implicating the crucial role of Wnt signaling in regulating both reactionary and reparative dentin formation (Babb et al., 2017). Likewise, wnt signaling modulation via its ligands and components affects pulp cell behavior and subsequent pulp response to injury. A major canonical ligand, Wnt3a, enhances mineralization and tertiary dentin formation *in vivo* (Sukarawan et al., 2023). A few other bioactive molecules have been reported to induce tubular dentin regeneration through activating Wnt signaling. Lithium chloride (LiCl) activates the canonical Wnt signaling pathway through inhibition of GSK-3β (Klein and Melton, 1996). Application of LiCl on the exposed rat pulp generated a dentin bridge with tubular characteristic of normal dentin, while the control group showed osteodentin formation (Ishimoto et al., 2015). Later, LiCl was incorporated into surface pre-reacted glass fillers, which reinforced the generation of reparative dentin that had a continuous structure with that of the original dentin (Ali et al., 2019). Semaphorin 3A is another factor reported to enhance reparative dentin formation when applied to the pulp exposure site. Expressed in neural crest-derived tissue, Semaphorin 3A activates odontoblast differentiation *in vitro* and tubular dentin regeneration *in vivo*, possibly through the canonical Wnt signaling (Yoshida et al., 2016).

### 1.3 Peptide and protein therapeutics

Enamel matrix protein and their derivatives have been studied for their tissue regeneration and remineralization potentials (Bächli et al., 2019). Amelogenin is the major constituent of enamel matrix proteins and is known to facilitate enamel remineralization and odontogenic differentiation of pulp cells, hence enhancing tertiary dentin formation (Al-Hezaimi et al., 2011). Its derivative peptide QP-5 has been found to promote mineralization of human dental pulp cells (hDPCs) *in vitro* and tertiary dentin formation in *in vivo* pulp capping rat model (Peng et al., 2021). Altering the hydrophilic C-terminal of amelogenin led to a synthetic peptide TVH-19 that retains hydrophobic and antimicrobial traits (Wang et al., 2017). It was demonstrated to induce mineralization of hDPCs *in vitro* and tertiary dentin formation in *in vivo* indirect pulp capping rat model (Han et al., 2021). However, the newly generated tertiary dentin showed rather repaired structures, lacking dentinal tubules.

A number of other bioactive molecules have been investigated as therapeutic pulp capping agents and reported to induce tertiary dentin formation either in their protein or oligopeptide forms. Protein S100-A7 is a bioactive molecule released from dentin matrix components after being digested by matrix metalloproteinase-20 (MMP-20). It has been shown to promote formation of tertiary dentin with tubule structures in the predentin area and odontoblast-like cells in *in vivo* pulp exposure rat models (Komichi et al., 2019). A recent study identified functional peptides derived from protein S100-A8, which accelerated tertiary dentin formation in a rat direct pulp capping model (Watanabe et al., 2023). Leptin, which is a peptide hormone secreted from adipose tissues, was also found to induce angiogenesis and dentinal bridge formation with tubule structures, alongside the osteodentin in *in vivo* direct pulp capping rat models (Choi et al., 2019). As a multifunctional glycoprotein found in plasma and the extracellular matrix of tissues, vitronectin plays a significant role in various physiological processes, including cell adhesion, migration, and proliferation. Its derivative synthetic oligopeptide, VnP-16, was used as a direct capping agent in a rat pulp exposure model, generating thicker and more homogenous tertiary dentin than MTA or BMP-2. No cellular entrapment was observed in the newly mineralized tissue, while tubular structure similar to physiologic dentin was evident (Park et al., 2022).

An epithelium-derived factor that is also a member of the non-collagenous dentin matrix proteins has been reported to induce odontoblast differentiation *in vitro* and tubular dentin regeneration *in vivo*. Copine7 (CPNE7) is a phospholipid binding protein initially identified in preameloblast-conditioned medium (PA-CM) from murine apical bud cells, which induces odontogenic differentiation of human dental pulp stem cells (hDPSCs) (Lee et al., 2011). Autophagy induced by CPNE7 removes lipofuscin, which accumulates with aging and hinders cellular functions, from mature odontoblasts and thus reactivates physiologic activities of odontoblast. It also enhances expression of TAU, a microtubule-associated protein, and this leads to cytoskeletal re-organization and sequential odontoblast process elongation, which are essential in physiologic dentin formation (Park et al., 2021). When the recombinant form of CPNE7 was applied to exposed dentin close to pulp in indirect pulp capping (IPC) beagle model, formation of tertiary dentin with dentinal tubules continuous with the previous dentin, was verified (Choung et al., 2016). Likewise, in dentin hypersensitivity beagle model, where dentinal tubules were exposed shallowly, CPNE7 induced tertiary dentin formation at dentin-pulp boundary and reduced microleakage (Park et al., 2019). Copine7-derived functional peptide (CPNE7-DP), a newly developed oligopeptide reproducing the functions of CPNE7 was shown to directly pass through cell membrane and induce tertiary dentin formation in both direct and indirect pulp capping models (Lee et al., 2020). Clinical applications of CPNE7-DP as a therapeutic agent for dentinal defects should be further evaluated.

### 1.4 Novel biomaterials and techniques

Bioactive glasses comprised of silica (SiO_2_), sodium oxide (Na_2_O), calcium oxide (CaO), and phosphorus pentoxide (P_4_O_10_) in specific proportions are actively studied biomaterials in bone and dentin formation research. Bioactive glass compounds have been demonstrated as a potential substitute for MTA due to their abilities to induce reparative dentin formation following direct pulp capping. When a newly developed bioactive glass-based cement was used as a pulp capping agent, thick reparative dentin with dentinal tubules was formed in the pulp exposure sites (Hanada et al., 2019).

Other bioceramics and polymers have been shown to serve as bioactive agents in dentin regeneration studies. Treated dentin matrix hydrogel (TDMH), which is a tissue-engineered combination of human treated dentin matrix and hydrogel, was found to promote dentin formation with homogenous tubular structure and well-organized odontoblasts *in vivo* (Holiel et al., 2021). Three-D-printed microgels supplemented with dentin matrix molecules (DMM) also induced formation of tertiary dentin and organized pulp tissue in *in vivo* direct pulp capping rat models (Cunha et al., 2023). Tertiary dentin with tubular organization was observed alongside atubular structures and new blood vessel formation, implying its potential to be used as a therapeutic agent for vital pulp therapy.

A novel bioactive adhesive monomer, CMET, was developed by combining calcium salt and 4-methacryloxyethyl trimellitate acid (4-MET), and its odontogenic induction capacity was tested in *in vivo* direct capping rat model. The results indicated that CMET produced tertiary dentin with distinguishable dentinal tubule patterns (Qiu and Saito, 2021). Surface pre-reacted glass ionomer (S-PRG) fillers have also been shown to stimulate tertiary dentin formation with tubular structures in *in vivo* direct pulp capping rat models. In this study, scanning electron microscopy (SEM) was employed to analyze microstructure of the newly formed hard tissues instead of histological staining. As mentioned earlier, combination of S-PRG fillers and lithium chloride induced tertiary dentin without defects in *in vivo* direct pulp capping rat model (Ali et al., 2019).

Recently, synthetic compounds capable of releasing nitric oxide (NO), termed nitric oxide donors (e.g., NOC-18), have found applications in various biological areas. Nitric oxide is a short-lived inert gas molecule that is known to mediate inflammatory responses and modulate cell proliferation and migration during tissue repair. It was demonstrated that applying exogenous nitric oxide NOC-18 in dentin exposure sites accelerated the formation of reparative dentin retaining the regular tubule structure (Sonoda et al., 2018). Additionally, pulp exposure sites were repaired by dentin bridge that was lined by an odontoblast layer when NOC-18 was used as a direct pulp capping agent in a beagle model (Alnour et al., 2023).

## 2 Discussion

**FIGURE 1:**
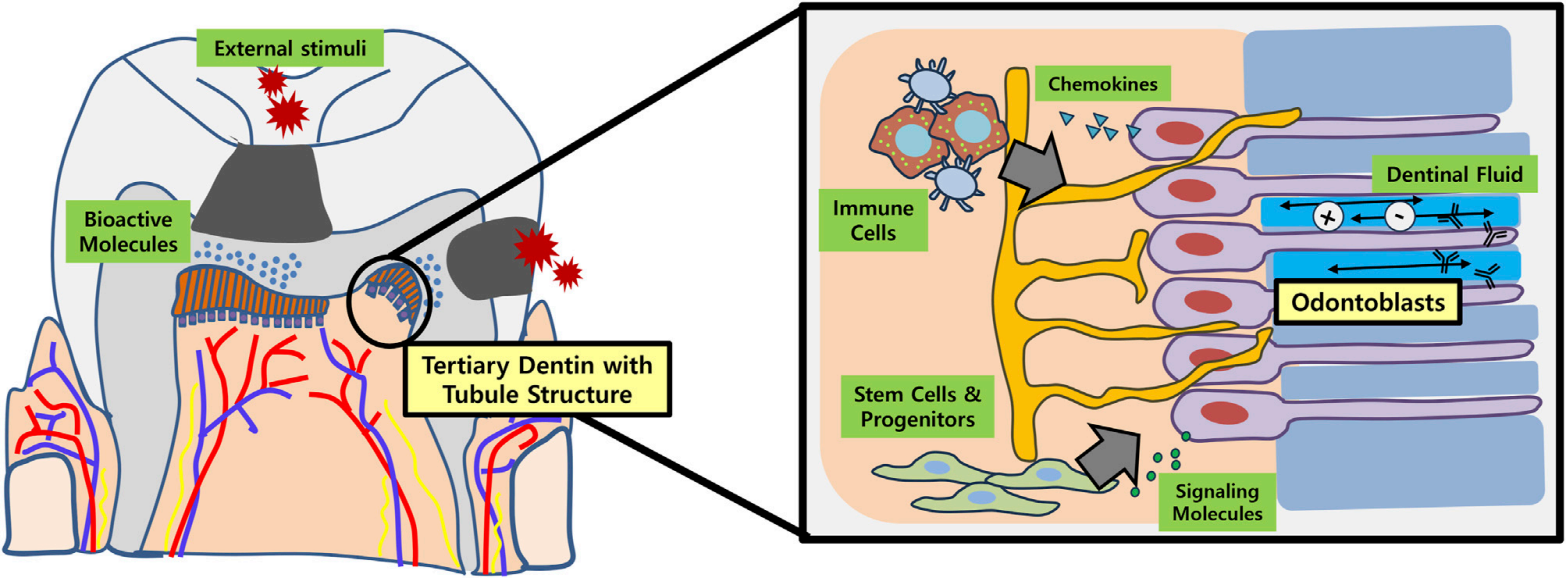
Schematic illustration of tubular dentin regeneration and its significance. Application of bioactive molecules onto the site of dentinal defects (e.g., dental caries) may enable regeneration of physiologic tubular dentin aligned by an odontoblast layer. These newly formed or re-activated odontoblasts play important roles in recruiting immune cells and stem/progenitor cells in the dental pulp by secreting chemokines and various signaling molecules, respectively. In conjunction with dental afferent nerve fibers, the odontoblasts also participate in sensory perception. Furthermore, the dentinal fluid contains ions and immunoglobulins that are essential for re-mineralization and overall defense of dentin against external stimuli.

The primary objective for dentin regeneration is to restore the physiologic functions of original dentin (Figure 1). In this regard, tubular structure with presence of dentinal fluid and odontoblast process is crucial (da Silva et al., 2020). Not only does regenerated tubular dentin render sensory capabilities and immune defense, but induces re-innervation of pulp and new dentin production in case of pathologic conditions like dental caries (Huang et al., 2020). Many of the biomolecules aimed at dentin regeneration, however, only resulted in formation of bone-like reparative dentin without dentinal tubules, lacking structural and functional integrities with the original tissue.

Implementing bioactive molecules capable of regenerating physiologic dentin into actual clinical settings is another consideration. For instance, dental caries, which is one of the most common chronic oral diseases, requires restoration of the destructed tissue. Several lines of evidence indicate that an inflamed pulp differs from a normal pulp in its response to growth factor application and healing capacity (Rutherford and Gu, 2000; Cooper et al., 2014; El karim et al., 2021). Yet in most literatures reviewed, the regenerative potentials of bioactive molecules were assessed with capping models in freshly exposed healthy dental pulps. Inflammation-associated chemoattractant C5a formed in carious pulp was shown to induce pulp progenitor cell migration (Chmilewsky et al., 2013). Moreover, polarization of anti-inflammatory macrophage by Wnt/β-catenin signaling promotes reparative dentin formation (Neves et al., 2020). Taking into account the characteristics of diseased pulpal condition, topical application of bioactive molecules onto the site of dentinal defect can be attempted as a therapeutic regimen. Therapeutic potential of the cell-penetrating peptide CPNE7-DP have recently been evaluated *in vivo* in an induced rat caries model (Gug et al., 2022). Likewise, bioactive molecules can expectedly eliminate the need for extensive and invasive excavation of carious lesions, rendering minimally invasive dentistry more plausible.

Dentin hypersensitivity is another pathologic condition that requires dentin recovery for relief of symptoms. Pre-existing desensitizers, however, provide mere temporary effects as they tend to break away when encountered with mechanical forces or acidic environment. In this aspect, physiologic dentin regeneration not only recovers impermeability of original dentin with new hard tissue barrier, but also provides additional dentin sealing with intratubular mineralization (won Kim and Park, 2017).

Regeneration of dentin-pulp complex as a whole further expands the scope of future research. While both cell-based and cell-free therapies show potential for dentin-pulp regeneration, scaffold in which biomolecules are delivered is of main concern for clinical implementation. It is necessary for bioactive molecules to be released gradually at a defined rate and duration in order to have sustained effects. Without scaffolds or carriers however, they exhibit a release pattern of a high initial burst followed by a quick decline (Kim, 2017). Therefore, controlled release of biomolecules assisted by adequate biodegradable scaffolds will enhance the effects of tissue regeneration. In case where pulp tissue is removed by pulpotomy or pulpectomy as in regenerative endodontic protocol, endogenous growth factors have been the main driving force for tissue regeneration. Perhaps the simultaneous treatment of exogenous biomolecules with adequate scaffolds can be investigated in subsequent research.

**FIGURE 2:**
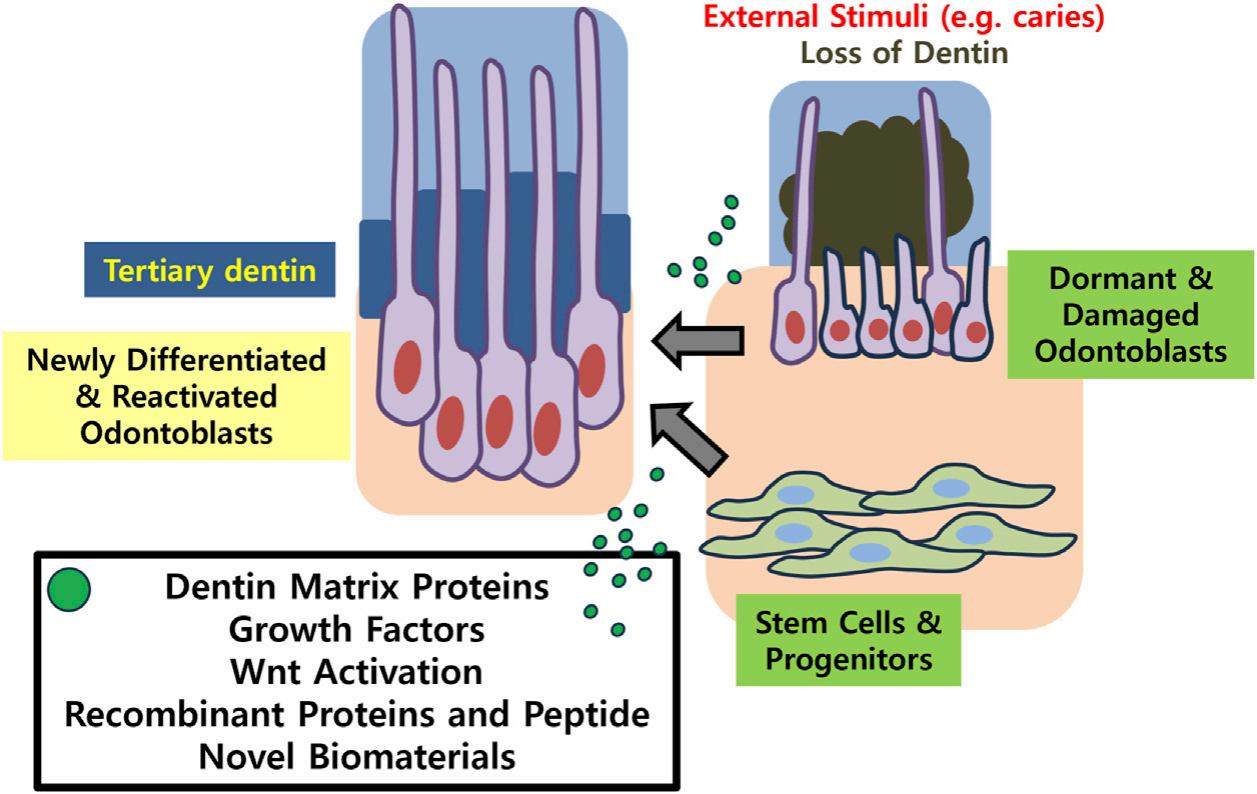
Schematic illustration summarizing the key concepts of the present review.

The limitations of current research involve that histologic observation only roughly hints at the presence of dentinal tubules. The regularity of the structure and whether the cells retain the characteristics of true odontoblasts should further be examined with electron microscopy and expression of specific marker proteins may be analyzed. Evaluating the physiologic functions of dentin should follow the structural observation, in order to confirm the nature of the newly formed dentin. Another limitation rises from the fact that previous *in vivo* studies have been performed predominantly in murine models. Experiments conducted under controlled conditions in small animals do not always translate to treatments for humans. Therefore, future directions for *in vivo* studies should point to larger mammalian models, while the next steps will involve conducting clinical trials in humans and seeking for drug development. Ultimately, clinical translation of bioactive molecule research should assist patients to maintain their natural dentition, with its physiological functions restored (Figure 2).
